# The impact of tumour size on the probability of synchronous metastasis and survival in renal cell carcinoma patients: a population-based study

**DOI:** 10.1186/1471-2490-14-72

**Published:** 2014-08-31

**Authors:** Johann P Ingimarsson, Martin I Sigurdsson, Sverrir Hardarson, Vigdis Petursdottir, Eirikur Jonsson, Gudmundur V Einarsson, Tomas Gudbjartsson

**Affiliations:** 1 Departments of Urology and Surgery, Memphis, Tennessee, USA; 2 Pathology, Landspitali University Hospital, Reykjavik, Iceland; 3 Faculty of Medicine, University of Iceland, Reykjavik, Iceland; 4 Dartmouth-Hitchcock Medical Center, Lebanon, New Hampshire, USA

**Keywords:** Renal cell carcinoma, Size, Metastasis, Survival

## Abstract

**Background:**

The observed low metastatic potential and favorable survival of small incidentally detected renal cell carcinomas (RCCs) have been a part of the rationale for recommending partial nephrectomy as a first treatment option and active surveillance in selected patients. We examined the relationship between tumor size and the odds of synchronous metastases (SMs) (primary outcome) and disease specific survival (secondary outcome) in a nationwide RCC registry.

**Methods:**

Retrospective study of the 794 RCC patients diagnosed in Iceland between 1971 and 2005. Histological material and TNM staging were reviewed centrally. The presence of SM and survival were recorded. Cubic spline analysis was used to assess relationship between tumor size and probability of SM. Univariate and multivariate statistics were used to estimate prognostic factors for SM and survival.

**Results:**

The probability of SM increased in a non-linear fashion with increasing tumor size (11, 25, 35, and 50%) for patients with tumors of ≤4, 4.1-7.0, 7.1-10.0, and >10 cm, respectively. On multivariate analysis, tumor size was an independent prognostic factor for disease-specific survival (HR = 1.05, 95% CI 1.02-1.09, p < 0.001), but not for SM.

**Conclusion:**

Tumor size affected the probability of disease-specific mortality but not SM, after correcting for TNM staging in multivariate analysis. This confirms the prognostic ability of the 2010 TNM staging system for renal cell cancer in the Icelandic population.

## Background

The ability to detect subclinical small renal cell carcinomas (RCCs) has greatly improved due to increased usage of abdominal imaging for unrelated disease [1]. This has altered the therapeutic approach to RCCs and over the past decade, nephron-sparing surgery (NSS) has replaced radical nephrectomy as the preffered treatment for RCCs less than 7 cm in size, when technically feasible, as suggested by both the European and American Guidelines [2,3]. These guidelines further recommend active surveillance or other minimally invasive treatments (i.e. radioablation or cryotherapy) as an alternative to surgery for patients with significant co-morbidities or advanced age. This is in part based on the assumption that the biological behaviour of smaller tumours is more benign than that of larger tumours, after observations of a low rate of metastasis and better survival of patients with smaller tumours [2-4].

The importance of knowing the exact size in prediction of outcome has been a matter of debate in the literature. While the study by Klatte et al., which focussed on tumours smaller than 4 cm, did not find a significant relationship between tumor size and the probability of SM and survival, numerous other studies have supported such a correlation [5-11]. Several studies have evaluated various size cut-off points to improve prognostication, but there have been conflicting results [12]. The 2010 revision of the TNM system emphasized size by subdividing the T2 stage into T2a and T2b catergories at the 10-cm cut-off point, although subsequent publications have questioned any prognostic difference between the two new stages [13,14].

Most studies that have investigated the relationship between size and SM have been based on data from single or multiple institutions. Importantly two population-based databases are available: the Surveillance, Epidemiology, and End-Results Database (SEER) and the National Swedish Kidney Cancer Quality Register (NSKCR) [6,7,15,16]. While covering larger populations, these databases did not offer a central review of staging or pathology, risking inter observer discrepancies with regards to stage and histology.

We therefore conducted a nationwide study using a centralized RCC database and a revised histological classification to test the relationship between RCC size and the probability of SM (primary outcome) and DSS (secondary outcome).

## Methods

We conducted a retrospective nationwide study that included all patients diagnosed with RCC in Iceland between January 1, 1971 and December 31, 2005. The Icelandic Cancer Registry and centralized pathology and clinical registries of all hospitals in Iceland were cross-checked with the inclusion list, giving a 100% match.

Clinical information was obtained from the medical records, including age, gender, symptoms, diagnostic work-up, and treatment. An incidental diagnosis was defined as tumours detected with imaging techniques or during surgery for reasons other than those related to clinical signs caused by renal tumours. In surgical cases, routine lymphadenectomy was not performed, but enlarged lymph nodes were usually removed. Postoperative follow-up was performed at each hospital or at outpatient clinics for the first 1–2 years. Follow-up thereafter for recurrence was per physician preference and not standardized. A chest X-ray was performed in all patients preoperatively, together with a computed tomography (CT) and/or ultrasound (US) of the liver when these tests became available. CT scans of the chest, brain, bone scans, magnetic resonance imaging (MRI), or cavography were performed selectively. PET scan was not available in Iceland during the study period.

Two of the authors, both consulting pathologists (S.H. and V.P), reviewed haematoxylin and eosin-stained slides, using accepted criteria by WHO and the Heidelberg classification for stratifying the histological subtypes [17]. The following pathology findings were recorded: Fuhrman nuclear four-grade scale, lymph node status, invasion of vessels, adrenals, and Gerotas fascia. As imaging modalities varied during the study period, tumour size was determined from the pathology specimens as the greatest diameter in mm. In the non-surgically treated cases, size, as measured by radiologists from CT scans, US, or intravenous pyelograms, was used. The spread of tumours was assessed using the most recent version of the TNM classification system and with stage grouping (I–IV) as proposed by AJCC [17,18]. Synchronous metastases were defined as metastases present at or within three months of the diagnosis of the primary kidney tumour.

All the patients were followed up for survival by using data from the updated National Population Registry, and they were assigned a date and cause of death (as registered on the death certificate) or identified as living on 31 December 2009. Median follow-up of survival was 4.5 years (range 0–34 years), and none of the patients were lost to follow-up.

Chi-square test and Mann–Whitney U test were used to compare univariate proportions and means, respectively. A p-value of < 0.05 was considered to be statistically significant. To graphically demonstrate a relationship between tumour size and probability of SM, we used cubic spline analysis—which makes no assumption about the relationship between parameters and is entirely data-driven. Independent prognostic factors for the presence of SM were assessed with logistic regression. The model included all univariate variables with p < 0.1. This included age, gender, calender year of diagnosis, tumor size, incidental diagnosis, histological subtype, laterality, estimated sedimentation rate (ESR) and pT stage. Since pT stage is highly dependant on size, analysis for SM were performed both with and without pT stage.

Cox proportional hazards analysis was used to determine independend predictors of and disease-specific survival (DSS). Cox proportional hazards analysis was used to determine independend predictors of disease-specific survival (DSS), using data from all patients with sufficient histology and staging data (n = 794). The model included all univariate variables with a p-value of < 0.1, including size, group TNM-stage and Fuhrman grade, ESR levels, incidental diagnosis, and calendar year of diagnosis. In the multivariate analysis for SMs, the pT stage rather than the TNM group stage was used, since the presence of metastases was included in the TNM group subset. Size was considered a continuous variable in all multivariate analysis.The statistical software package R, version 2.10.1, was used for all analyses (the R Foundation, Vienna, Austria).

This study was approved by the National Bioethics Committee and the Icelandic Data Protection Agency. As individual patients were not identified, individual consent for the study was waived.

## Results

Altogether, 913 patients were diagnosed with RCC during the 35 year study period, but 116 patients were excluded; 65 because detailed information on tumor size was lacking and 51 cases that only were clinically diagnosed as RCC (histology missing). This left 794 with histopathologically confirmed disease that were used for calculations. Nephrectomy was performed in 702 out of 794 cases (4.1% of them partial) with 92 patients being treated non-surgically. No patients were treated with cryothearpy or radioablation, as these techniques were only available after 2006 in Iceland.

Patient demographics and treatment are shown in Table 1. The ratio of males to females was 1.5, the average age was 65 years, and 29% of the tumors were detected incidentally.

**Table 1:** Patient characteristics

**Average age (range)**	**64.0 ± 12.9 (17–93)**
Male: female ratio	1.6
Right kidney, n (%)	413 (59)
Incidental detection, n (%)	150 (21)
First treatment	
Radical nephrectomy, n (%)	673 (84)
Partial nephrectomy, n (%)	29 (4)
Non surgical on no treatment, n (%)	92 (12)

### Size and synchronous metastases

Mean tumor size was 7.2 ± 3.8 cm (median 6.7, range 0.3–30.0). Tumors diagnosed in the first 5 years were larger (8.4 cm) than those diagnosed in the last 5 years (6.5 cm) (p < 0.01 for trend). The proportion of patients with confirmed pT stages 1, 2, 3, and 4 were 35%, 17%, 39%, and 4%, respectively. Altogether, 247 patients (30%) were diagnosed with SMs, with lungs (n = 121), bones (n = 80), and liver (n = 51) being the most common metastatic sites.

**Figure 1:**
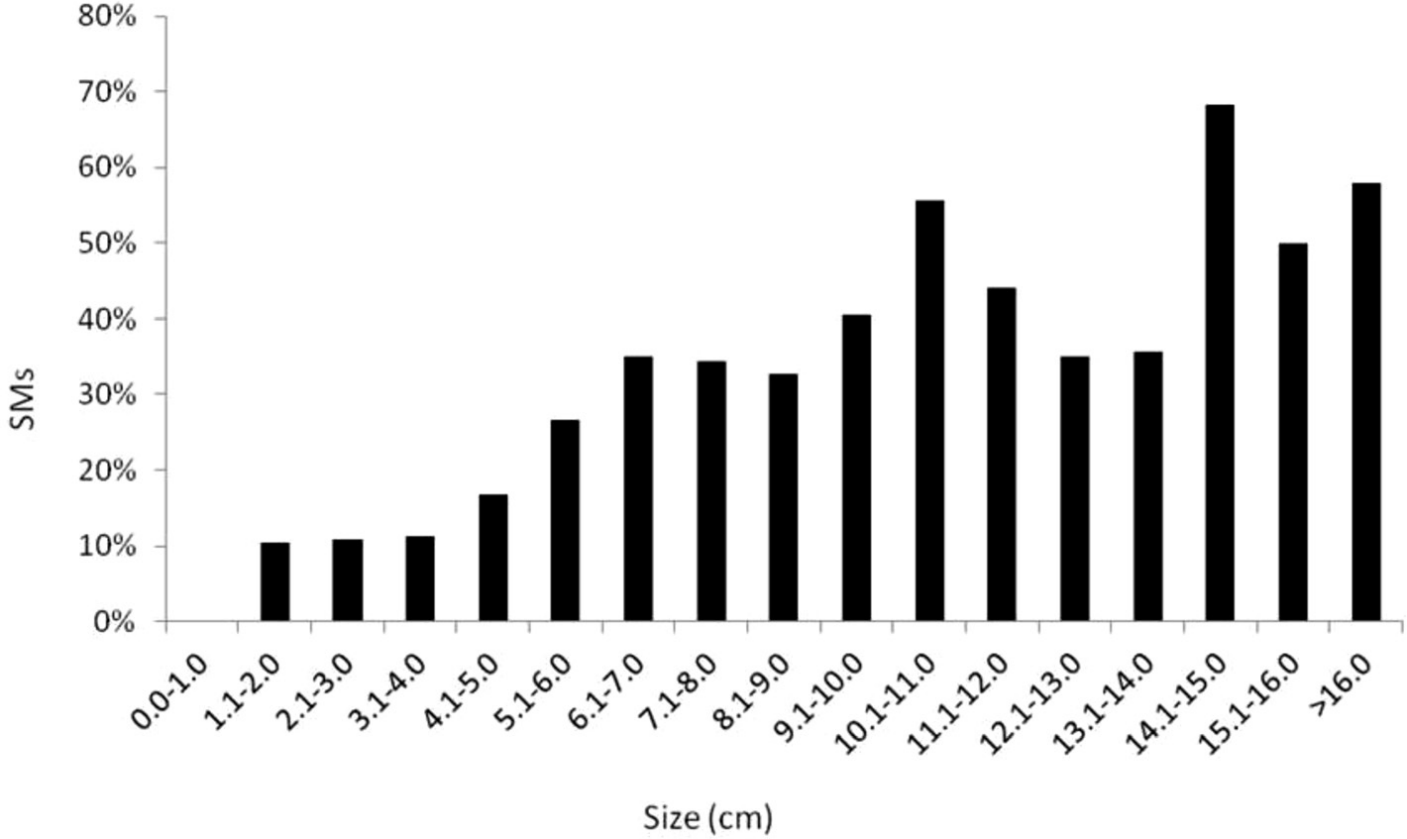
**Proportion of synchronous metastases as stratified by 1-cm tumor size intervals.** Tumors above 16 cm (n = 18) have been pooled, as there were less than 2 cases in each 1-cm category.

**Figure 2:**
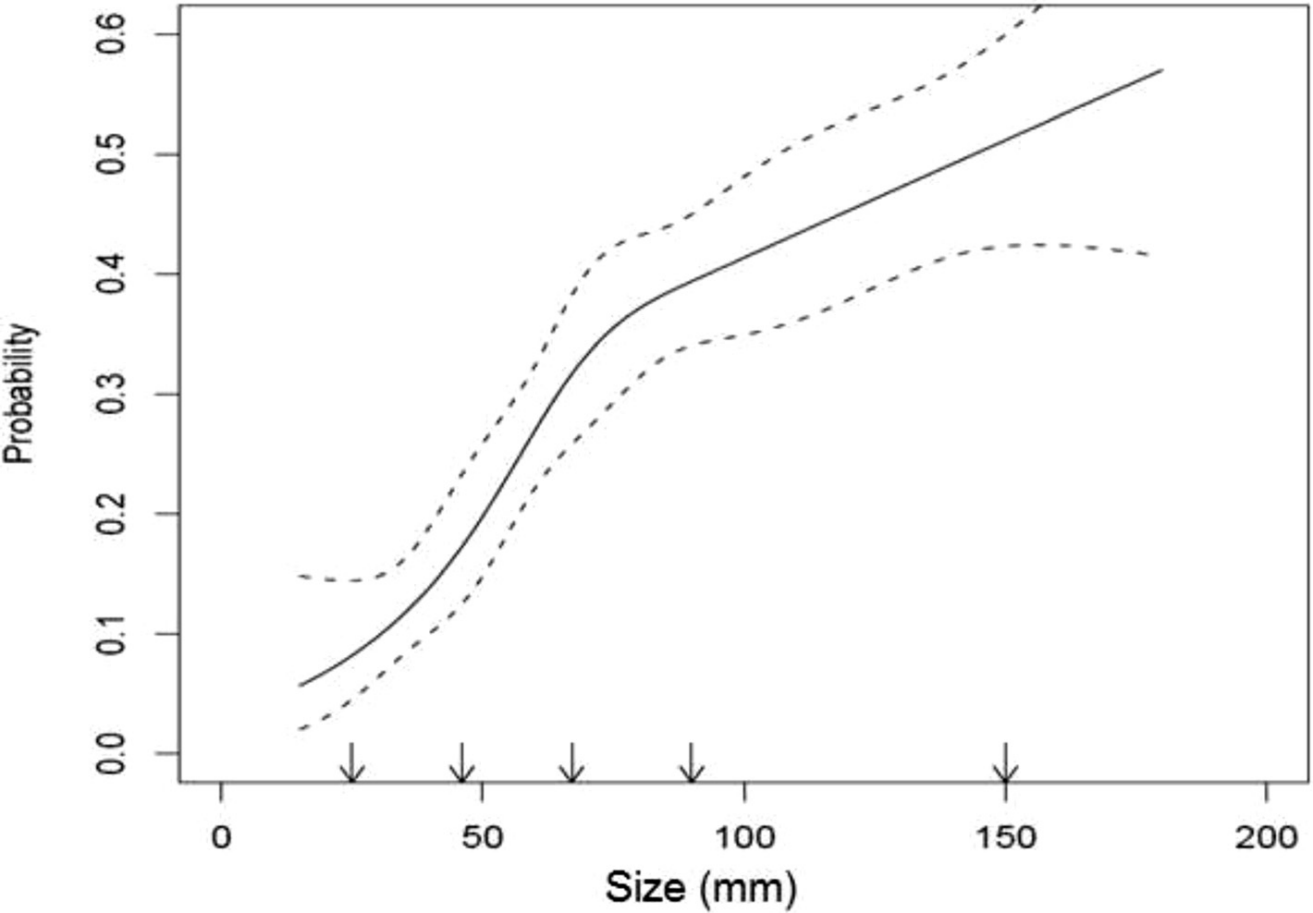
**A cubic spline graph showing the relationship between tumour size and the probability of synchronous metastases in RCC patients dignosed in Iceland, 1971–2005.** Dotted lines show 95% CI.

When pathologically confirmed tumors were classified into 1-cm size categories, there was a positive correlation between increasing size and the probability of SM (Figure 1) (R^2^ = 0.80, p < 0.0001). This correlation was retained in a sub analysis of localized tumors (pT1 and pT2) (R^2^ = 0.83, p < 0.0001), but not when the pT3 or pT4 tumors were analyzed (R^2^ = 0.58, p = 0.12). Figure 2 shows a cubic spline analysis of the relationship between size and the probability of SM.

Table 2 shows a comparison of patients with and without SM. Patients with SM had significantly larger tumors (mean 9.0 vs. 6.5 cm) (p < 0.001), higher T stage, higher Fuhrman grade, higher ESR, and earlier calendar year of diagnosis. Furthermore, they were less frequently diagnosed incidentally and more often had tumor in the left kidney. There were, however, no significant differences in gender, age, or histological subtype. The proportion of SM for tumours less than 4.0 cm was 11% (20/188), but 7% when only tumors detected after 1990 were included (90% of them being diagnosed incidentally).

**Table 2:** Comparison of clinicopathological factors for RCC patients diagnosed with and without synchronous metastases (SMs) in Iceland, 1971–2005

	**N**	**SM (%)**	**Without SM (%)**	p-value*
**Mean age**	794	65 (±13)	65 (±13)	0.87
**Gender**				
Male	481	137 (29)	344 (71)	0.81
Female	313	87 (28)	226 (72)	
**Year of diagnosis**				< 0.001
1971–1975	65	23 (35)	45 (65)	
1976–1980	65	22 (36)	41 (64)	
1981–1985	92	36 (39)	56 (61)	
1986–1990	103	40 (38)	64 (62)	
1991–1995	127	32 (25)	95 (75)	
1996–2000	139	25 (18)	114 (82)	
2001–2005	202	44 (22)	158 (78)	
**Diagnosis**				< 0.001
Incidental	236	14 (6)	222 (94)	
Symptomatic	558	207 (37)	351 (63)	
Laterality**				< 0.001
Right kidney	427	94 (22)	333 (78)	
Left kidney	359	122 (34)	237 (66)	
**Mean ESR**		29.7 (±32)	59.1 (±39)	< 0.001
**Tumor size by category**				< 0.001
0.1–4.0	177	19 (11)	158 (89)	
4.1–7.0	252	64 (25)	188 (75)	
7.1–10.0	226	80 (35)	146 (65)	
10.1–13.0	76	35 (46)	41 (54)	
13.1–16.0	46	24 (53)	22 (47)	
> 16.0	17	10 (61)	7 (39)	
**T stage**				< 0.001
T1a	154	6 (4)	148 (94)	
T1b	140	12 (9)	128 (91)	
T2a	92	16 (17)	76 (82)	
T2b	51	14 (36)	37 (64)	
T3	328	125 (38)	203 (62)	
T4	30	11 (63)	19 (47)	
**Histological RCC subtype**				0.13
Clear cell	705	194 (28)	511 (72)	
Papillary	65	14 (22)	41 (78)	
Chromophobe	15	1 (7)	14 (93)	
Other	9	3 (33)	5 (66)	
**Nuclear grade**				< 0.001
I - II	449	66 (15)	383 (85)	
III - IV	345	147 (43)	198 (57)	

*Mann–Whitney test for means, Chi-square test for trends.

**Excluding 8 bilateral cases.Number of patients is given with percentage in parentheses, except for ESR and age where mean is given together with standard deviation.

A multivariate logistic regression analysis revealed that symptomatic diagnosis, elevated ESR, left kidney tumor and a size increment of 1 cm were independent prognostic factors for SM (Table 3). In a model including tumor stages, size was not retained as an independent prognostic factor. However, symptomatic presentation, elevated ESR and left kidney tumor continued to be independent prognostic factors. When included, higher tumour stage was the strongest predictor in all models.

**Table 3:** Multivariate analysis of the importance of size for the rate of synchronous metastases in patients diagnosed with RCC in Iceland, 1971–2005

	**Odds ratio**	**95% CI**	**p**
Tumor size (per cm)	1.09	1.04-1.16	0.001
Age	1.00	0.98-1.01	0.9
Female gender	0.87	0.57-1.30	0.49
Later diagnostic year (per decade)	1.02	0.91-1.13	0.94
Incidental diagnosis	0.22	0.11-0.41	<0.001
ESR (each 1 mm/h)	1.15	1.09-1.21	<0.001
Left side	1.67	1.12-2.50	0.013
Histology			
Clear cell	1	-	-
Chromophobe	1.37	0-infinity	0.98
Papillary	0.93	0.42-2.03	0.81
Other/unknown	1.27	0.11-14.71	0.5

ESR and Size are continuous varibles.

### Size and survival

There was significantly better survival in patients with smaller tumors (p < 0.001, log rank test). DSS at 5 years was 86% for tumors of < 4 cm, 72% for tumors between 4 and 7 cm, 53% for tumors between 7 and 11 cm, and 32% for tumors larger than 11 cm.

In a univariate analysis, less favorable DSS was related to the following factors: increasing tumor size, higher group stage and Fuhrman grade, high ESR levels, presentation with symptoms, and year of diagnosis. In the Cox analysis shown in Table 4, TNM group stage, Fuhrman nuclear grade, calendar year of diagnosis, and incidental detection were still independent predictors of DSS. Size remained a statistically significant albeit small predictor (HR = 1.05, CI 1.02–1.09, p <0.001) when TNM group stage was added to the multivariate model.

A subcategory of patients with histopathologically confirmed and organ confined (T1/T2,N0,M0) RCC (without synchronous metastasis) underwent nephrectomy (n = 370); 6% of them with partial nephrectomy. Sixty four of the 370 patients (17.4%) ultimately died from RCC, 5–371 (mean 100, medial 70) months after surgery. Patient characteristics and univariate analysis are shown in Table 5. A multivariate logistic regression analysis revealed that only year of diagnosis (OR 0.93 for each calendar year), elevated ESR (OR 1.01 per 1 mm/hour) and a size (OR 1.21 per one cm increment, 95% CI 1.03-1.58, p = 0.02) were independent prognostic factors for DSS in this subcategory of patients. If the multivariate model also included tumor stage, size fell short of being retained as an independent prognostic factor (OR 1.17, 95% CI 0.99-1.39, p = 0.053).

**Table 4:** Multivariate analysis of the predictors for disease specific survival in patients diagnosed with RCC in Iceland, 1971–2005

	**Hazard ratio**	**95% CI**	**p-value**
Tumor size (cm)	1.05	1.02–1.09	0.01
Later diagnostic year (by decades)	0.90	0.85-0.95	0.01
Incidental detection	0.74	0.52–1.03	0.08
Left sided tumour	1.14	0.91-1.41	0.24
TNM group stage			
1	1	-	-
2	2.34	1.36-3.99	<0.001
3	3.54	2.21-5.71	<0.001
4	13.16	8.14-21.28	<0.001
Fuhrman nuclear grade			
1	1	-	-
2	0.79	0.41-1.54	0.49
3	1.10	0.56-2.13	0.78
4	1.74	0.86-3.49	0.12
Histology			
Clear cell	1	-	-
Chromophobe	0.13	0.01-0.97	0.046
Papillary	0.88	0.52-1.46	0.61
Other/unknown	1.61	0.39–6.64	0.51

**Table 5:** Comparison of clinicopathological factors of patients with organ confined RCC without synchronous metastases who underwent nephrectomy by cause of death

	**N**	**Death from RCC (%)**	**Did not die from RCC (%)**	p-value*
**Mean age**	370	62 (±13)	62 (±12)	0.62
**Gender**				
Male	227	41 (18)	186 (82)	0.67
Female	143	23 (16)	120 (84)	
**Year of diagnosis**				< 0.001
1971–1975	26	8 (31)	18 (79)	
1976–1980	27	9 (33)	18 (67)	
1981–1985	45	10 (22)	35 (88)	
1986–1990	36	10 (28)	26 (82)	
1991–1995	59	12 (20)	47 (75)	
1996–2000	79	9 (11)	72 (89)	
2001–2005	96	6 (8)	90 (92)	
**Diagnosis**				< 0.001
Incidental	165	18 (11)	147 (89)	
Symptomatic	205	46 (22)	159 (78)	
Laterality**				0.004
Right kidney	205	28 (14)	177 (86)	
Left kidney	165	36 (22)	129 (78)	
**Mean ESR (mm/h)**		21.0 (±24)	38.8 (±40)	< 0.001
**T stage**				< 0.001
T1a	144	7 (5)	137 (95)	
T1b	124	22 (18)	102 (82)	
T2a	76	24 (32)	52 (68)	
T2b	26	11 (42)	15 (58)	
**Tumor size (cm)**		5.1 (±2.9)	7.6 (±3.1)	< 0.001
**Histological RCC subtype**				0.21
Clear cell	315	56 (18)	256 (82)	
Papillary	42	8 (19)	34 (81)	
Chromophobe	11	0 (0)	11 (100)	
Other	2	0 (0)	2 (0)	
**Nuclear grade**				< 0.001
I - II	299	40 (14)	259 (85)	
III - IV	71	24 (34)	47 (66)	

Number of patients is given with percentage in parentheses, except for ESR and age where mean with standard deviation is given.

*Mann-Whitney test for means, Chi-square for trends.

## Discussion

Our results show that increasing size of RCC tumours is associated with a higher probability of SM and worse survival in an unselected nationwide RCC registry. Tumour size remained an independant predictive factor for DSS after multiple corrections, albeit with a limited additional prognostic value when the TNM group stage was added to the model. While tumor size did predict SM in multivariate analysis, it did not independently predict the probability of SM when the T stage was added to the model.

Our results add to the findings from studies using the SEER and the NSKCR databases, which were also population based patient cohorts [6,7,15]. The strength of our study is that the data include all patients diagnosed in a whole country over a 35-year period. This eliminates the risk of inclusion bias or referral bias. Other strengths are the centrally reviewed histology and complete follow-up with regard to survival. We intentionally excluded clinically diagnosed RCCs without histopathologic confirmation as these cases may have included benign kidney tumors and thus falsely improved the survival. It could also be argued that by excluding the clinically diagnosed cases, the RCC patients with the worst prognosis may have been omitted. However, including the clinically diagnosed patients from the multivariate analysis did not significantly change our main findings, neither for SM-rate or survival. A weakness of the study is the retrospective design, relatively small number of patients, with differences in the quality and quantity of staging and metastatic work-up over time, as well as changes in treatment options being offered. Further, in our study, average size is larger and incidental diagnosis less frequent than obtained in more contemporary series and may not reflect the more recent increase in incidentalomas.

The correlation between size and the probability of SM was strong in our data (Figures 1 and 2). Our findings are in line with the study by Kunkle et al. where tumour size independently predicted the odds of SM [9]. More recently, an almost linear relationship between size and SM for tumours ≤ 7 cm was described by Gudmundsson et al. in the NSKCR study [7]. In addition, based on the SEER database, Lugezzani et al. reported a similar relationship for T1 tumours, and Nguyen et al., found a sigmoidal relationship between size and SM that was comparable to the findings in the present study [6,15].

The proportion of SM in the present study was higher than reported in other clinical studies, most of them contemporary series, with SM rates ranging between 0.1% and 7% [10,19-21]. In population databases, the reported proportion of SM from the SEER database ranged from 3% to 6% and in the NSKCR study it was 7% [6,7,15]. This is similar to the SM-rate (7%) found in our patients diagnosed after 1990. Unlike NSKCR, we did not evaluate the relationship between size, SM, and lymph node involvement, as lymph nodes were not systematically sampled or removed.

For tumors between 4.0 and 7.0 cm, we found the relationship between size and SM rate to be virtually linear. This is in line with most previous studies [6,9,15]. For tumours larger than 7.0 cm, earlier reports have shown that further increase in size adds less to metastatic potential [6,15]. While the shape of our cubic spline might suggest the same trend, the confidence interval also widens with increasing size, making it difficult to support such statements.

Despite the seemingly strong relationship between size and SM, it is noteworthy that the association is not statistically retained in a multivariate analysis after correcting for pT stage, grade, incidental detection, laterality, and other factors that were significant in the univariate analysis. At the same time, size clearly correlated with DSS in the Cox multivariate analysis. It has previously been shown in multivariate analyses not including stage, that size is a significant predictor of DSS, disease-free survival, and SM, with HR and OR of 1.2 or less [22,23]. Karakiewitz et al. included TNM stage in the multivariate analysis of 2,245 patients and found that knowing the exact size added 3.7% to the predictive accuracy for SM and only 0.8% to the predictive accuracy for DSS (2002 version) [24]. This, as well as the strong predictive value of of pT stage for SM and TNM stage for DSS in the present study, appears to confirm the value of the most recent (7th) version of the TNM-staging system.

## Conclusion

We have shown in a nationwide cohort that tumour size correlates strongly with the rate of SM and is an independent prognostic factor of long-term survival after correcting for confounding variables. Tumor size therefore aids in prognostication, and is an addition to that predicted by the TNM-staging system, however, the difference is small.

## Competing interests

The authors declare they have no competing interests.

## Authors’ contributions

JPI and TG designed the study and wrote the manuscript. JPI, TG and GVE obtained infromation from charts SH and VP performed the pathological review, MIS performed the statistical analysis. EJ, GVE, SH, VP and MIS reviewed and edited the manuscript. All authors read and approved the final manuscript.

## Pre-publication history

The pre-publication history for this paper can be accessed here:

http://www.biomedcentral.com/1471-2490/14/72/prepub
