# Erratum to: The impact of tumour size on the probability of synchronous metastasis and survival in renal cell carcinoma patients: a population-based study

**DOI:** 10.1186/1471-2490-15-5

**Published:** 2015-02-16

**Authors:** Johann P Ingimarsson, Martin I Sigurdsson, Sverrir Hardarson, Vigdis Petursdottir, Eirikur Jonsson, Gudmundur V Einarsson, Tomas Gudbjartsson

**Affiliations:** Departments Urology and Surgery, Landspitali University Hospital, Reykjavik, Iceland; Department of Pathology, Landspitali University Hospital, Reykjavik, Iceland; Faculty of Medicine, University of Iceland, Reykjavik, Iceland; Dartmouth-Hitchcock Medical Center, Lebanon, New Hampshire

## Text

In the original article [1] the list of authors were incorrectly affiliated. Please find the corrected list below.

### Author list

Johann P Ingimarsson^1,4*^, Martin I Sigurdsson^1^, Sverrir Hardarson^2^, Vigdis Petursdottir^2^, Eirikur Jonsson^1^, Gudmundur V Einarsson^1^ and Tomas Gudbjartsson^1,3^

### Author affiliations

^1^Departments Urology and Surgery, Landspitali University Hospital

^2^Department of Pathology, Landspitali University Hospital

^3^University of Iceland, Faculty of Medicine, Reykjavik, Iceland

^4^Dartmouth-Hitchcock Medical Center, Lebanon, New Hampshire

We would like to apologize for this error and for any inconvenience this may have caused.
